# Supplementary figures and images for: Cell-autonomous GABAARs are essential for NMDAR-mediated synaptic transmission, LTP, and spatial memory
Source: EMBO Rep. 2025 Jul 30;26(18):4456–76. doi: 10.1038/s44319-025-00538-x (PMC12457689; doi:10.1038/s44319-025-00538-x)

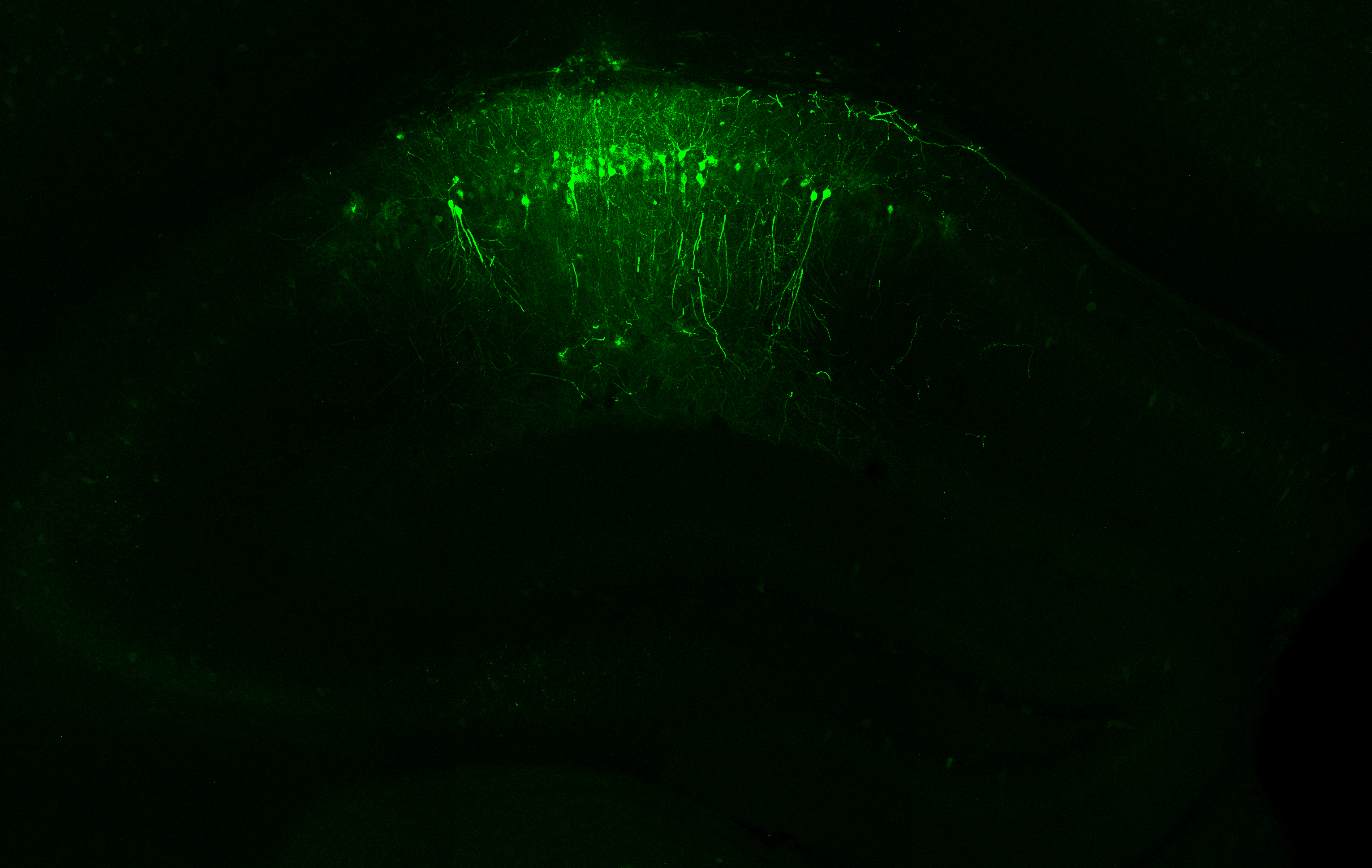

Supplement: Supplementary file 2 — Source data Fig. 1 [file 44319_2025_538_MOESM2_ESM.zip › Figure 1/Figure 1D.jpg]
