# Supplementary material for: Cell-autonomous GABAARs are essential for NMDAR-mediated synaptic transmission, LTP, and spatial memory
Source: EMBO Rep. 2025 Jul 30;26(18):4456–76. doi: 10.1038/s44319-025-00538-x (PMC12457689; doi:10.1038/s44319-025-00538-x)
Supplement: Supplementary file 8 — Expanded View Figures [file 44319_2025_538_MOESM8_ESM.pdf]

Expanded View Figures

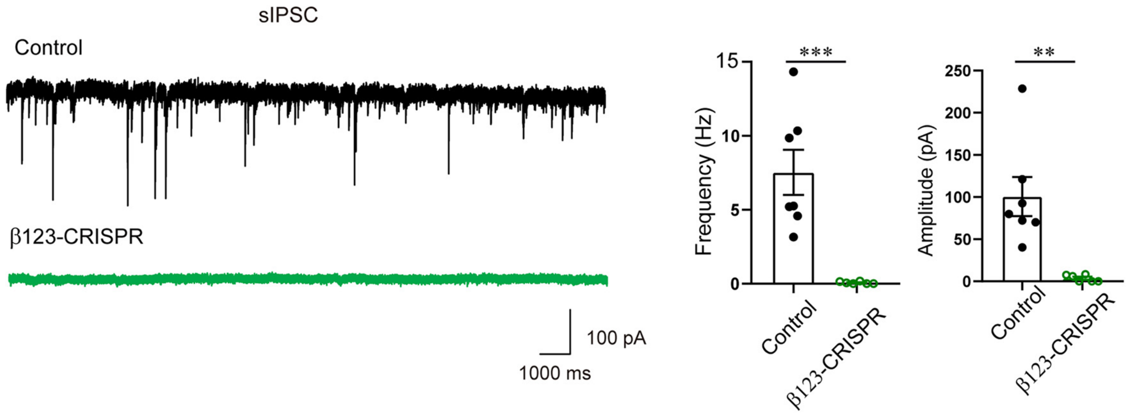

Figure EV1. Genetic deletion of $GABA_AR$ β1, β2, and β3 subunits via β123-CRISPR eliminated spontaneous IPSCs in hippocampal CA1 neurons expressing β123-CRISPR compared to control, related to Fig. 1.

Bar graphs indicate mean ± SEM, $n = 7$ for control, $n = 6$ for β123-CRISPR; $^{***}P = 0.0009$, $^{**}P = 0.0028$, unpaired t test.

© The Author(s)

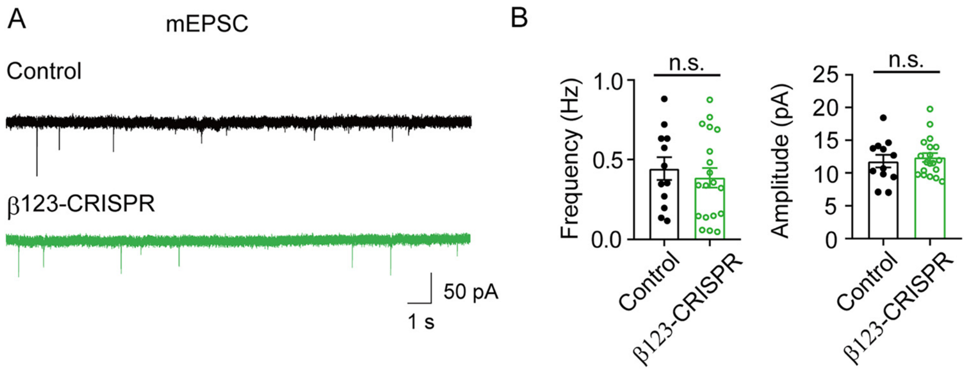

Figure EV2. Neither the amplitude nor the frequency of AMPAR-mediated mEPSCs showed a significant change in neurons expressing β123-CRISPR, related to Fig. 2.

(**A**) Representative traces of AMPAR-mediated mEPSCs recorded from control and β123-CRISPR expressing neurons. (**B**) Quantification of mEPSC amplitude and frequency. Bar graphs indicate mean ± SEM. Frequency: control vs β123-CRISPR, 0.44 ± 0.07 Hz vs 0.39 ± 0.06 Hz, $P = 0.55$; Amplitude: control vs β123-CRISPR, 11.79 ± 0.96 pA vs 12.34 ± 0.67 pA, $P = 0.63$; $n = 12$, 19, $N = 3$ for each group, n.s., not significant, unpaired t test.

 © The Author(s)

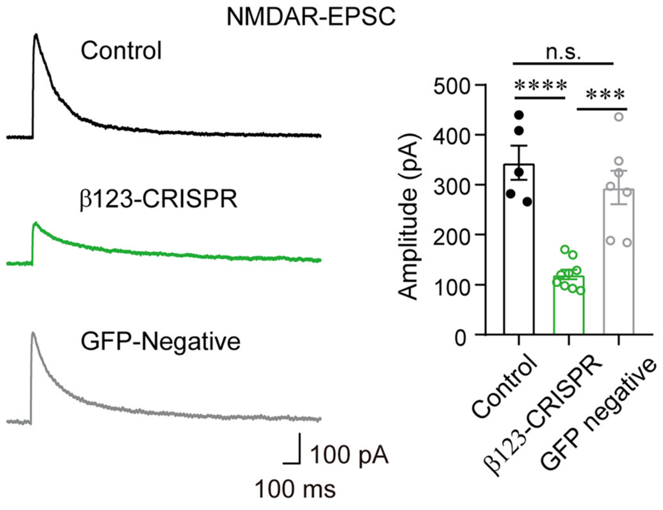

Figure EV3. NMDAR transmission remains unaltered in GFP-negative neurons in β123-CRISPR mice, related to Fig. 2.

Bar graphs indicate mean ± SEM, $n = 5$ for control, $n = 9$ for β123-CRISPR, $n = 7$ for GFP-negative neurons in β123-CRISPR mice; ***$P = 0.0002$, ****$P < 0.0001$, n.s., not significant, $P = 0.5105$, one-way AVOVA with Bonferroni's test.

© The Author(s)

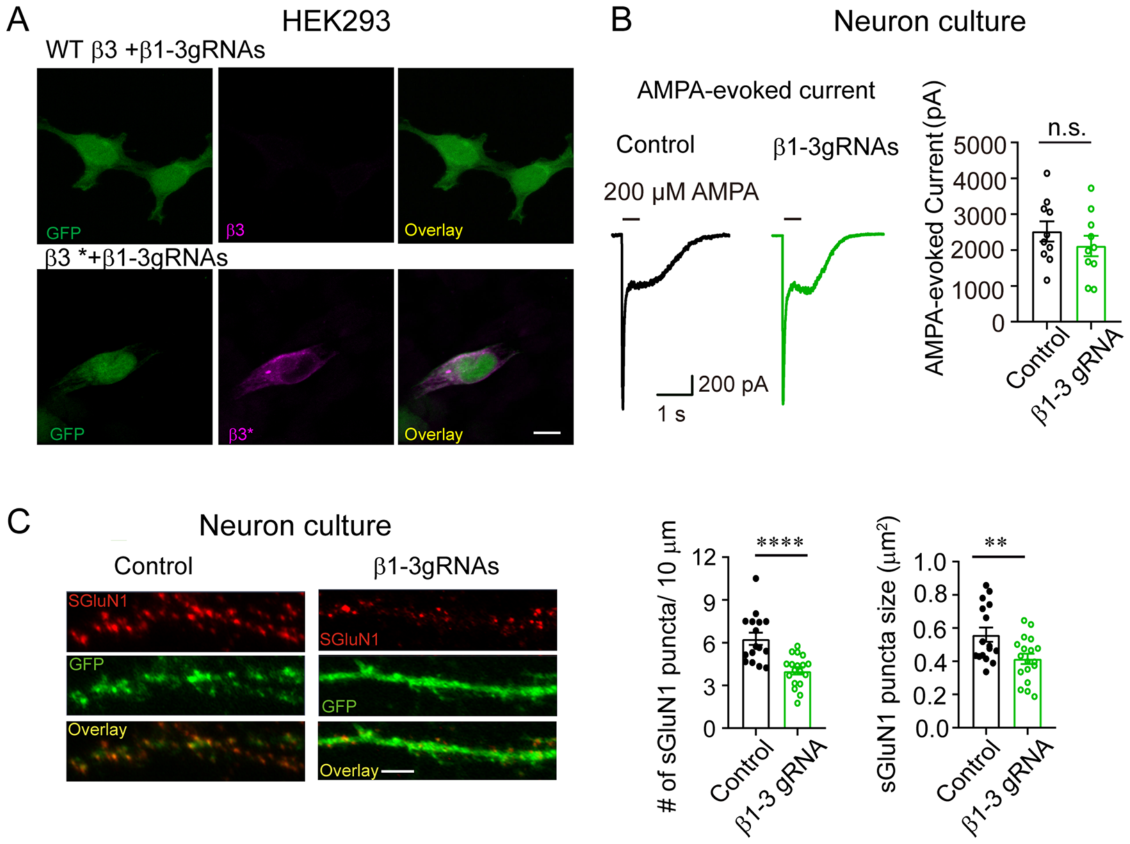

Figure EV4. β1-3 gRNA maintains gRNA-Resistant β3 (β3*) expression, preserves AMPA-evoked currents, but reduces surface GluN1, related to Fig. 3.

(**A**) Representative images showed that β1–3 gRNA failed to diminish the expression of the gRNA-resistant-β 3* (β3*) in HEK293T cells. Scale bar, 2 μm. (**B**) Loss of $GABA_A$Rs in neurons expressing β1–3 gRNAs didn't significantly affect AMPA-evoked whole-cell currents in hippocampal neuronal cultures (Bar graphs indicate mean ± SEM, $n = 10$, $N = 3$, $P = 0.3244$, unpaired t test). Bars indicate AMPA applications for AMPA-evoked whole-cell currents. (**C**) Loss of $GABA_A$Rs in neurons expressing β1–3 gRNAs significantly reduced surface GluN1 puncta (red) in hippocampal neuronal cultures. Representative images of surface GluN1 in neurons expressing control-GFP or β1–3 gRNAs (left). (Right) Bar graphs showed the quantitation of surface GluN1 puncta density and size in neuronal dendrites. Bar graphs indicate mean ± SEM, $n = 16$, 18 respectively, $N = 3$, $^{****}P < 0.0001$, $^{**}P = 0.0089$, unpaired t test). Scale bar, 5 μm.

 © The Author(s)

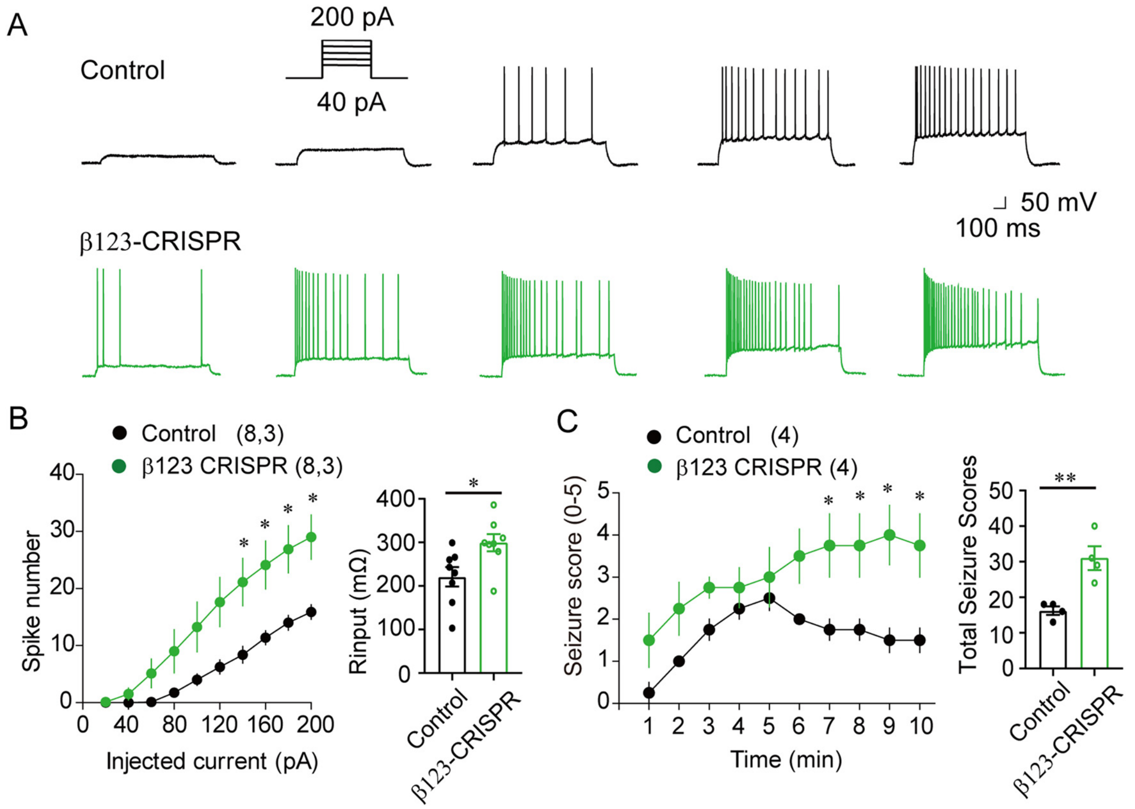

Figure EV5. Genetic deletion of $GABA_A$Rs in β123-CRISPR mice induced enhanced neuronal excitability and hippocampal hyperexcitability, related to Fig. 4.

(**A**) Sample action potential responses to step current injections (40, 80, 120, 160 and 200 pA; 1,000 ms) in control neurons (black) or β123-CRISPR neurons (green). (**B**) Left: summary graph showing that deletion of $GABA_A$Rs increased the excitability of CA1 pyramidal neurons (Line graphs indicate mean ± SEM, $^*P = 0.0324$, $n = 8$, $N = 3$, two-way repeated-measures ANOVA followed by Sidak's multiple comparisons test at each intensity). Right: Bar graph shows that input resistance is significantly enhanced in neurons lacking $GABA_A$Rs. $^*P = 0.02$, $n = 8$. unpaired *t* test, Error bars represent SEM. (**C**) Time course of average seizure scores induced by PTZ injection (40 mg/kg) (Line graphs indicate mean ± SEM, $^*P = 0.01$, $n = 4$ male mice/group, unpaired *t*-test). Behavioral responses were scored every 1 min for 10 min after the PTZ injection. Bar graph showing total seizure scores (Bar graphs indicate mean ± SEM, $^{**}P = 0.0059$, $n = 4$, unpaired *t* test).

© The Author(s)
